# Relationship between Smartphone Addiction and Sleep Satisfaction: A Cross-Sectional Study on Korean Adolescents

**DOI:** 10.3390/healthcare10071326

**Published:** 2022-07-17

**Authors:** Eonho Kim, Kihyuk Lee

**Affiliations:** 1 Department of Physical Education, Dongguk University, Seoul 04620, Korea; eonkim@dongguk.edu; 2 Department of Sport Culture, Dongguk University, Seoul 04620, Korea

**Keywords:** smartphone addiction, smartphone usage time, sleep satisfaction, sleep quality, Korea Youth Risk Behavior Web-Based Survey, adolescents

## Abstract

The purpose of this study was to analyze the relationship between smartphone addiction and sleep satisfaction in 54,948 Korean adolescents. This study utilized the Korea Youth Risk Behavior Web-Based Survey (KYRBS). The dependent variable was sleep satisfaction. Independent variables were smartphone addiction level and usage time. Gender, school grade, stress, depression, regular physical activity (PA), asthma, allergic rhinitis, and atopic dermatitis were selected as confounding variables. A chi-squared test, logistic regression, and independent t-test were performed for data analysis. As a result of the chi-squared test, sleep satisfaction showed significant relationships with all confounding variables (all *p* < 0.001). As a result of adjusting all confounding variables, sleep satisfaction of smartphone normal users was significantly higher (odds ratios: 1.372, *p* < 0.001) than that of high-risk users with smartphone addiction. Smartphone users with a daily smartphone usage time from 2 h to 8 h a day were 1.096–1.347 times (*p* = 0.014 to *p* < 0.001) more likely to be satisfied with their sleep than smartphone users with a daily smartphone usage time over 8 h, who were unsatisfied with their sleep. The group that was not satisfied with their sleep had a significantly higher average daily smartphone usage time and total score on the smartphone addiction scale than the group that was satisfied with their sleep (both *p* < 0.001). In conclusion, it will be necessary to manage the use of smartphones to improve the sleep satisfaction of Korean adolescents.

## 1. Introduction

Sleep is a basic requirement for human survival. It is very important for restoring tired bodies and minds and leading a normal life. Sufficient sleep plays an important role in maintaining the homeostasis of the central nervous system and human immune function [1]. Insufficient sleep causes symptoms such as memory loss, decreased cognitive function, and fatigue [2]. Despite the importance of sleep, most adolescents experience insufficient sleep qualitatively and quantitatively. According to previous reports [3], 69.7% of Korean adolescents answered that their sleep time was insufficient, while only 30.3% of adolescents were satisfied with their sleep time. Their average weekly sleep time was six hours and twelve minutes. This is less than 8 to 10 h a day, the recommended sleep standard time for adolescents presented by the American Academy of Sleep Medicine [4]. Insufficient sleep negatively affects adolescent growth, emotion, and immunity [5]. It shows a high association with childhood obesity [6]. Adolescents who continuously experience sleep disorders show increases in cortisol secretion [7]. This reduces memory and leads to a lack of concentration, which adversely affects their academic performance [8]. Healthy sleep habits in adolescence are very important because insufficient sleep during adolescence is likely to have a negative effect on adulthood [9].

The number of smartphone users is increasing due to increases in efficiency and comfort. However, excessive smartphone use has many side effects, such as stress and obesity [10,11]. Another problem with excessive mobile phone use is increased impulsive behavior [12]. It is associated with mechanisms involved in the ability to control behavior, which can potentially lead to smartphone addiction [13]. According to a survey on smartphone addiction in 2020 [14], 19.3% of the total population are potential risk users of smartphone addiction and 4.0% are high-risk users in Korea. Adolescents lack the ability to control impulsive behavior [15]. They are reported to be more susceptible to smartphone addiction than other age groups, with 30.8% of Korean adolescents being potential risk users and 5.0% being high-risk smartphone users in 2020. The increase in the potential risk of smartphone addiction among adolescents is related to psychopathological problems such as depression and anxiety, as well as academic achievement and life satisfaction [10,13,16]. Moreover, the number of adolescents at potential risk of smartphone addiction in 2020 due to the recent COVID-19 pandemic had increased by 5.6% compared to that in 2019 [17].

Meanwhile, several studies have reported that the prevalence of smartphones and the development of mass media hinder adolescents’ sleep [18,19,20]. A previous study has analyzed the relationship between sleep satisfaction and smartphone addiction among young adults aged 18 to 30 in the UK and reported that 39% of young adults are addicted to smartphones and that smartphone addiction is related to a lack of sleep, independently of smartphone usage time [21]. However, this cannot be applied to Korean adolescents because their lifestyles and cultural environment are different from those of western young adults. Meanwhile, Kim et al. (2016) have reported that the higher the smartphone addiction score, the lower the sleep quality of university students [22]. However, no study has reported the effect of smartphone addiction on the sleep satisfaction and sleep time of immature adolescents. Moreover, studies analyzing the relationship between smartphone addiction and sleep so far have not considered the various variables affecting sleep. Therefore, it is necessary to determine the effects of the smartphone addiction level of Korean adolescents on sleep satisfaction and sleep time considering various variables.

Thus, the purpose of this study was to analyze the relationship between smartphone addiction, smartphone usage time, and sleep satisfaction among Korean adolescents using the 16th Korea Youth Risk Behavior Web-Based Survey (KYRBS). Results of this study are expected to provide basic data for decreasing the risk of smartphone addiction and improving the sleep satisfaction of Korean adolescents.

## 2. Materials and Methods

### 2.1. Research Subjects and Data Collection Methods

This study used the 16th KYRBS conducted by the Korea Disease Control and Prevention Agency (KDCA). The KYRBS is an anonymous, self-reported online survey that has been conducted annually since 2005 to identify middle and high school students’ health behaviors. It consists of 103 items in 16 areas, such as life habits, mental health, diseases, smartphone addiction, and sleep satisfaction. This is a government-approved statistical survey (Approval No. 11758) that has been conducted annually since 2005. The KYRBS is conducted via the standardized operational procedures of the KDCA to ensure data quality. Prior to conducting the survey, the KDCA conducts training for sampled schools between April and May each year to ensure standardized data collection of the survey. A trained teacher explains the purpose of the survey and the process of participation and then students fill out anonymous self-management web-based questionnaires in a school computer lab with Internet access. It takes approximately 40 min for students to complete the questionnaire. Details of the KYRBS are described in a previous study [23]. The 16th KYRBS period was from 3 August 2020 to 13 November 2020. Sampling and recruitment were targeted at all students in the classes that were selected as sample classes through population stratification, sample distribution, and sampling stages. A total of 57,925 students from 800 schools (400 middle schools and 400 high schools) were possible participants. The students excluded from the survey were those with long-term absence on the day of the survey, special children, and students with text disabilities. Lastly, 54,948 students participated in this study, showing a participation rate of 94.9%. Our data represented 2,631,459 middle and high school students in the Republic of Korea.

### 2.2. Questionnaire Content and Variable Definition

#### 2.2.1. Independent Variables

The independent variables of this study were the smartphone addiction scale and smartphone usage time. Smartphone addiction is not a recognized clinical diagnosis. In this study, smartphone addiction was defined by referring to previous studies [24,25]. Smartphone addiction was classified by summing the scores of ten related questions in the questionnaire. Detailed components of this questionnaire are shown in Table 1. Each question was evaluated on a 4-point Likert scale. In the response to each question, the maximum of each score was 4 points, with 4 points for a response of “very much”, 3 points for “yes”, 2 points for “not”, and 1 point for “not at all”. Question scores were summed. The total possible score was 40 points. Depending on the total score, subjects were classified into a general user group (total sore < 22 points), a potential-risk user group (total score = 23 to 32 points), and a high-risk user group (total score ≥ 33). Cronbach’s alpha coefficient was 0.914 in the questionnaire reliability analysis. In addition, in this survey, smartphone usage time was investigated for 7 days and divided into weekdays and weekends. The following formula was used in this study to calculate smartphone average usage time for 7 days: ((Weekdays usage time ∗ 5) + (Weekends usage time ∗ 2))/7. The calculated smartphone usage time was classified into “under two hours”, “over two hours to under four hours”, “over four hours to under six hours”, “over six hours to under eight hours”, and “over eight hours” according to the original data usage guidelines of the KYRBS.

#### 2.2.2. Dependent Variable

The dependent variable of this study was sleep satisfaction. The question was, “Do you think the time you slept in the last seven days is enough to recover from fatigue?” Answers were “very satisfactory”, “satisfactory”, “just so-so”, “unsatisfactory”, and “very unsatisfactory”. Based on the original data usage guidelines of the KYRBS, answers were reorganized into two categories: yes (“satisfactory”) and no (“unsatisfactory”). Answers of “very satisfactory” and “satisfactory” were classified as “yes”. Answers of “just so-so”, “unsatisfactory”, and “very unsatisfactory” were classified as “no”. The method of evaluating sleep satisfaction used in this study has been frequently used in previous studies [26,27].

#### 2.2.3. Confounding Variables

Confounding variables were those that could affect sleep satisfaction and smartphone addiction. Gender, school grade, stress, depression, regular physical activity (PA), asthma, allergic rhinitis, and atopic dermatitis were used for the analysis. Among these confounding variables, gender and school grade were used without reconstruction. The question about stress was, “How much stress do you usually feel?” Answers of “I feel very much”, “I feel a lot”, and “I feel a little” were classified as “yes”, while answers of “I don’t feel much” and “I don’t feel any stress” were classified as “no”. The depression question was, “In the last 12 months, have you felt sad or hopeless enough to stop your daily life for two weeks?” Answers were classified as “yes” and “no”. Regular PA was classified as “yes” and “no”, where students who completed one or more of the following three physical activities were classified as “yes”: more than 5 days of moderate physical activity, more than 3 days of vigorous physical activity, and more than 3 days of strength exercise. The question about asthma, allergic rhinitis, and atopic dermatitis was “Have you been diagnosed with ‘asthma, allergic rhinitis, and atopic dermatitis’ by a doctor in the last 12 months?” Answers to this question were reconstructed into “yes” and “no”.

### 2.3. Statistical Analysis

Data were analyzed using the SPSS version 25.0 (SPSS Inc., Chicago, IL, USA). Data of this study were collected through complex sample designs. They were analyzed by applying strata, cluster, weight, and finite population correction according to the data usage guidelines of the KDCA. A chi-squared test (Rao–Scott) was conducted to elucidate the relationships between subjects’ confounding variables, smartphone addiction, and sleep satisfaction. Complex-samples logistic regression analysis was conducted to analyze the relationship between smartphone addiction level and sleep satisfaction according to smartphone usage time. Odds ratios (ORs) and 95% confidence intervals (CIs) were calculated by adding confounding variables. The difference between the average use time of a smartphone and the total score on the smartphone addiction scale according to sleep satisfaction was analyzed through an independent t-test. The statistical significance level was set at α = 0.05.

## 3. Results

### 3.1. General Characteristics of Survey Subjects and Results of Chi-Squared Test

The total number of subjects in this study was 54,948, including 16,824 students who were satisfied with their sleep and 38,124 students who were not satisfied with their sleep. The level of smartphone addiction (*p* < 0.001) and smartphone usage time (*p* < 0.001) were significantly different depending on sleep satisfaction. Table 2 shows differences in smartphone addiction and usage time based on sleep satisfaction. As a result of the chi-squared test to determine the relationship between sleep satisfaction and confounding variables (gender, school grade, stress, depression, regular physical activity, asthma, allergic rhinitis, and atopic dermatitis), all showed significant relationships with sleep satisfaction (all *p* < 0.001). Table 3 shows detailed chi-squared test results for the relationships between confounding variables and sleep satisfaction.

### 3.2. Relationship between Smartphone Addiction and Sleep Satisfaction

Table 4 shows the relationship between smartphone addiction and sleep satisfaction using the odds ratio. Compared to high-risk users who were unsatisfied with their sleep in Model 1, which was not adjusted, potential-risk users and normal users were 1.220 times (*p* = 0.003) and 2.123 times (*p* < 0.001) more likely to be satisfied with their sleep, respectively. In Model 2, which was adjusted for gender, grade, stress, depression, regular physical activity, and smartphone usage time as confounding variables, potential-risk users and high-risk users did not show a significant difference in sleep satisfaction (*p* = 0.701). However, normal users were 1.390 times (*p* < 0.001) more likely to be satisfied with their sleep than high-risk users. In Model 3, adjusted for asthma, allergic rhinitis, and atopic dermatitis in addition to the confounding factors adjusted in Model 2, potential-risk users and high-risk users showed no significant difference in sleep satisfaction (*p* = 0.828). However, normal users were 1.372 times (*p* < 0.001) more likely to be satisfied with their sleep than high-risk users.

### 3.3. Relationship between Smartphone Usage Time and Sleep Satisfaction

Table 5 shows the relationship between smartphone usage time and sleep satisfaction using the odds ratio. Compared to those who used smartphones for over 8 h and felt unsatisfied with their sleep in Model 1, those who used smartphones for under 2 h to under 8 h were 1.855–1.156 times (*p* < 0.001) more likely to be satisfied with their sleep. In Model 2 and Model 3, such odds ratios were 1.339–1.094 (*p* = 0.016~*p* < 0.001) and 1.347–1.096 (*p* = 0.014~*p* < 0.001), respectively.

### 3.4. Differences in Daily Average Smartphone Usage Time and Smartphone Addiction According to Sleep Satisfaction

**Figure 1:**
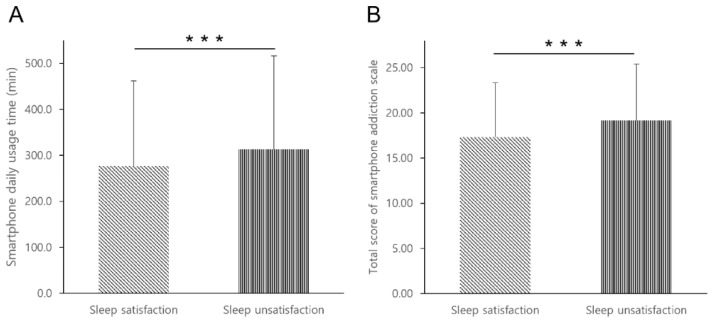
Differences in smartphone usage time and smartphone addiction according to sleep satisfaction: (**A**) daily average smartphone usage time; (**B**) total score on smartphone addiction scale; *** *p* < 0.001.

It was found that students who were not satisfied with their sleep had a significantly (*p* < 0.001) higher average smartphone usage time per day compared to those who were satisfied with their sleep. In addition, it was found that students who were not satisfied with their sleep had a higher total score on the smartphone addiction scale than those who were satisfied with their sleep (*p* < 0.001). Figure 1 shows differences in average smartphone daily usage time and total scores on the smartphone addiction scale according to sleep satisfaction.

## 4. Discussion

Although it has been reported that smartphone addiction and usage time have a negative effect on sleep satisfaction, previous studies have not considered the various variables that affect sleep. In this study, in order to consider various variables, the 16th KYRBS was used to analyze the difference in sleep satisfaction according to the level of smartphone addiction and usage time in Korean adolescents.

This study collected data from a large sample of Korean adolescents. Sleep satisfaction was significantly different depending on gender, grade, stress, depression, regular physical activity, asthma, allergic rhinitis, and atopy. The prevalence of smartphone addiction was analyzed using a verified and widely used smartphone addiction scale. Smartphone usage time was investigated. As a result, sleep satisfaction was significantly different depending on the level of smartphone addiction and the time of use. High-risk users and those who used smartphones for over 8 h accounted for the highest percentage of sleep dissatisfaction.

As a result of the cross-analysis of sleep satisfaction by selecting confounding variables that might affect sleep satisfaction, all confounding variables showed significant relationships with sleep satisfaction. Regarding sleep satisfaction according to gender, female adolescents had a higher percentage of sleep dissatisfaction than male adolescents (74.1% vs. 64.5%). This was similar to previous study comparing the difference in sleep satisfaction between men and women [28]. As for sleep satisfaction according to school grade, the 7th grade showed the highest percentage of sleep satisfaction, and the 12th grade showed the highest percentage of sleep dissatisfaction in the present study. This seems to be due to the lack of sleep time, because the higher the grade, the more time the participants spent preparing for college entrance. This result was the same as those reported in a previous study [29]. Moreover, adolescents who responded that they experienced stress and depression showed the highest percentage of sleep dissatisfaction. Notably, in addition to the possibility that smartphone addiction affects the quality of sleep, both smartphone addiction and sleep quality are likely to be caused by depression [30]. Accordingly, an analysis was conducted by setting depression as a confounding variable (Model 2 and 3), and, as a result, both smartphone usage time and the level of smartphone addiction affected sleep satisfaction. As for sleep satisfaction according to regular physical activity, adolescents who did not perform regular physical activity accounted for the highest rate of sleep dissatisfaction. For adolescents with asthma, allergic rhinitis, and atopic dermatitis, known to interfere with sleep, they had the highest rate of sleep dissatisfaction. A previous study reported that allergic rhinitis and atopic dermatitis negatively affect adolescents’ sleep satisfaction compared to those with asthma [27], but it is an advantage of our study that we examined the relationship between smartphone addiction and sleep satisfaction using these variables as confounding variables.

As a result of cross-analysis of the difference in sleep satisfaction according to smartphone addiction and usage time in this study, sleep satisfaction significantly differed according to the level of smartphone addiction and usage time. In Model 3, in the logistic analysis after adjusting for all confounding variables, sleep satisfaction was increased by 1.372 times for normal users compared to high-risk users with smartphone addiction. In the case of smartphone usage time, users who spent less than 8 h to less than 2 h increased their sleep satisfaction by 1.096 to 1.347 times compared to users who spent more than 8 h daily using smartphones. A previous study has already reported negative effects of screen time on sleep, especially smartphones, which are portable devices that are more likely to interfere with the quality or the amount of sleep [31]. Another previous study reported that using a smartphone for more than 60 min before bedtime increases the probability of poor sleep quality by 7.486 times [32].

In addition, excessive smartphone use has also been reported to be related to daytime fatigue, long sleep delays, and sleep time reduction [33,34]. The results of our study support previous studies showing that smartphone addiction has a detrimental effect on sleep [31,35]. This relationship was still significant after applying daily usage time and smartphone addiction level as confusion variables, respectively. These findings suggest that, similar to other addictions, usage time is a risk factor for smartphone addiction. It might be a component determining smartphone addiction, although it is not the only determinant. This result indicates that the relationship between smartphone addiction and sleep satisfaction is not simply due to smartphone usage time, as suggested by other studies [36]. Sleep dissatisfaction cannot be interpreted as simply being due to a decrease in sleep time and an increase in smartphone usage time.

Meanwhile, regular physical activity or moderate intensity of exercise can be one way to improve sleep satisfaction for Korean adolescents. Previous studies have reported that physical activity and exercise not only can lower the risk of smartphone addiction, but also can improve the quality of sleep [37,38,39]. Kim and Lee have reported that the risk of addiction to smartphones increases by 1.351–1.549 times if adolescents do not perform regular physical activity [25]. Another study has reported that higher levels of overall physical activity can improve both sleep quantity and quality [40]. However, since many previous studies have reported that excessive exercise can interfere with sleep, the intensity of exercise or performance time suitable for one’s physical fitness level should be considered when performing exercise to improve sleep satisfaction.

This study conducted a survey of 54,948 Korean adolescents by applying the complex sampling method and inferred results representing the total population of adolescents in Korea in 2020 by applying weights. Smartphone addiction and usage time showed close relationships with sleep satisfaction. However, this study has several limitations. Since our study was a cross-sectional study, we should consider the possibility of an inverse causal relationship among the results. In addition, it should be considered that this study relied on the subjective responses of participants for the measurement of sleep satisfaction. In the future, it is necessary to analyze the effects of smartphone addiction and usage time on objective sleep quality.

## 5. Conclusions

Sleep satisfaction differed according to the level of smartphone addiction and smartphone usage time. There was a difference in sleep satisfaction even after adjusting for all confounding variables, such as gender, grade, stress, depression, regular exercise, asthma, rhinitis, and atopic dermatitis, in Korean adolescents. The lower the level of smartphone addiction or the lower the usage time, the higher the probability of feeling satisfied with one’s sleep. To improve the satisfaction with sleep for Korean adolescents, it is necessary to decrease the risk of addiction to smartphones or reduce the usage time.

## Figures and Tables

**Table 1:** Components of the smartphone addiction scale for Korean adolescents.

Factor	Questions
Self-Control Failure	① Every time I try to reduce my smartphone time, it fails ② It is difficult to control the smartphone usage time ③ It is hard to keep proper usage time for smartphones
Salience	④ If a smartphone is next to me, it’s difficult to focus on other tasks. ⑤ Smartphone thoughts do not leave my mind ⑥ I strongly feel the urge to use my smartphone
Serious Consequences	⑦ I have had health problems because of smartphone use ⑧ I had a hard time fighting with my family because of my smartphone ⑨ I have experienced severe conflicts in my friends, colleagues or social relationships because of my smartphone ⑩ I have difficulties in carrying out work (study or job) due to smartphone

**Table 2:** General characteristics of participants according to sleep satisfaction.

	Total Users (*n* = 54,948)			x^2^ (*p*)
	Sleep Satisfaction (*n* = 16,824)	Sleep Unsatisfaction (*n* = 38,124)	Total (*n* = 54,948)	
Gender				
Male	9960 (35.5)	18,393 (64.5)	28,353 (100.0)	594.704
Female	6864 (25.9)	19,731 (74.1)	26,595 (100.0)	<0.001
School grade				
7th	4104 (41.2)	5901 (58.8)	10,005 (100.0)	1042.677
8th	3333 (34.6)	6231 (65.4)	9564 (100.0)	<0.001
9th	2916 (31.1)	6476 (68.9)	9392 (100.0)	
10th	2221 (24.5)	6686 (75.5)	8907 (100.0)	
11th	2317 (25.9)	6590 (74.1)	8907 (100.0)	
12th	1933 (23.7)	6240 (76.3)	8173 (100.0)	
Stress				
No	6112 (51.9)	5795 (48.1)	11,907 (100.0)	3208.805
Yes	10,712 (24.8)	32,329 (75.2)	43,041 (100.0)	<0.001
Depression				
No	14,218 (34.7)	26,890 (65.3)	41,108 (100.0)	1231.316
Yes	2606 (18.8)	11,234 (81.2)	13,840 (100.0)	<0.001
Regular physical activity				
No	9104 (28.5)	22,890 (71.5)	31,994 (100.0)	179.951
Yes	7720 (33.8)	15,234 (66.2)	22,954 (100.0)	<0.001
Asthma				
No	16,679 (30.8)	37,674 (69.2)	54,353 (100.0)	11.642
Yes	145 (24.3)	450 (75.7)	595 (100.0)	<0.001
Allergic rhinitis				
No	14,541 (31.6)	31,452 (68.4)	45,993 (100.0)	118.796
Yes	2283 (25.9)	6672 (74.1)	8955 (100.0)	<0.001
Atopic dermatitis				
No	15,913 (31.0)	35,470 (69.0)	51,383 (100.0)	39.345
Yes	911 (26.0)	2654 (74.0)	3565 (100.0)	<0.001

Values are *n* (%). Percentage is weighted.

**Table 3:** Differences in smartphone addiction and daily average smartphone usage time according to sleep satisfaction.

	Total Users (*n* = 54,948)			x^2^ (*p*)
	Sleep Satisfaction (*n* = 16,824)	Sleep Unsatisfaction (*n* = 38,124)	Total (*n* = 54,948)	
Smartphone addiction				
Normal user	13,759 (33.6)	27,414 (66.4)	41,173 (100.0)	645.733
Potential-risk user	2750 (22.5)	9392 (77.5)	12,142 (100.0)	<0.001
High-risk user	315 (19.2)	1318 (80.8)	1633 (100.0)	
Smartphone usage time				
Over 8 h	2159 (24.4)	6607 (75.6)	8766 (100.0)	440.627
Over 6 h to under 8 h	2122 (27.2)	5736 (72.8)	7858 (100.0)	<0.001
Over 4 h to under 6 h	4456 (29.6)	10,593 (70.4)	15,049 (100.0)	
Over 2 h to under 4 h	5611 (34.0)	10,994 (66.0)	16,605 (100.0)	
Under 2 h	2476 (37.5)	4194 (62.5)	6670 (100.0)	

Values are presented as *n* (%). Percentage is weighted.

**Table 4:** Logistic regression analysis of sleep satisfaction based on smartphone addiction.

	Model 1		Model 2		Model 3	
Smartphone Addiction	OR (95% CI)	*p*	OR (95% CI)	*p*	OR (95% CI)	*p*
High-risk user	Reference	-	Reference	-	Reference	-
Potential-risk user	1.220 (1.068–1.394)	0.003	1.028 (0.894–1.181)	0.701	1.016 (0.883–1.168)	0.828
Normal user	2.123 (1.871–2.410)	<0.001	1.390 (1.216–1.589)	<0.001	1.372 (1.199–1.568)	<0.001

OR: odds ratio; Model 1 was not adjusted; Model 2 was adjusted for gender, school grade, stress, depression, regular physical activity, and daily average smartphone usage time; Model 3 was adjusted for gender, school grade, stress, depression, regular physical activity, daily average smartphone usage time, asthma, allergic rhinitis, and atopic dermatitis.

**Table 5:** Logistic regression analysis of sleep satisfaction based on daily average smartphone usage time.

	Model 1		Model 2		Model 3	
Smartphone Usage Time	OR (95% CI)	*p*	OR (95% CI)	*p*	OR (95% CI)	*p*
Over 8 h	Reference	-	Reference	-	Reference	-
Over 6 h to under 8 h	1.156 (1.079–1.239)	<0.001	1.094 (1.017–1.178)	0.016	1.096 (1.018–1.180)	0.014
Over 4 h to under 6 h	1.299 (1.222–1.381)	<0.001	1.148 (1.076–1.225)	<0.001	1.151 (1.079–1.229)	<0.001
Over 2 h to under 4 h	1.590 (1.493–1.694)	<0.001	1.240 (1.159–1.326)	<0.001	1.244 (1.163–1.331)	<0.001
Under 2 h	1.855 (1.733–1.985)	<0.001	1.339 (1.245–1.439)	<0.001	1.347 (1.252–1.448)	<0.001

OR: odds ratio; Model 1 was not adjusted; Model 2 was adjusted for gender, school grade, stress, depression, regular physical activity, and level of smartphone addiction; Model 3 was adjusted for gender, school grade, stress, depression, regular physical activity, level of smartphone addiction, asthma, allergic rhinitis, and atopic dermatitis.

## Data Availability

Releasing of the data by the researcher is not legally permitted. All data are available from the database of the KDCA.
